# Inhibition of Cell Proliferation and Cell Death by Apigetrin through Death Receptor-Mediated Pathway in Hepatocellular Cancer Cells

**DOI:** 10.3390/biom13071131

**Published:** 2023-07-14

**Authors:** Pritam Bhagwan Bhosale, Hun Hwan Kim, Abuyaseer Abusaliya, Se Hyo Jeong, Min Yeong Park, Hyun-Wook Kim, Je Kyung Seong, Meejung Ahn, Kwang Il Park, Jeong Doo Heo, Young Sil Kim, Gon Sup Kim

**Affiliations:** 1 Department of Veterinary Medicine, Research Institute of Life Science, Gyeongsang National University, Jinju 52828, Republic of Korea; shelake.pritam@gmail.com (P.B.B.); shark159753@naver.com (H.H.K.); yaseerbiotech21@gmail.com (A.A.); tpgy123@gmail.com (S.H.J.); lilie17@daum.net (M.Y.P.); kipark@gnu.ac.kr (K.I.P.); 2 Division of Animal Bioscience & Intergrated Biotechnology, Jinju 52725, Republic of Korea; hwkim@gnu.ac.kr; 3 Laboratory of Developmental Biology and Genomics, BK21 PLUS Program for Creative Veterinary Science Research, Research Institute for Veterinary Science, College of Veterinary Medicine, Seoul National University, Seoul 08826, Republic of Korea; snumouse@snu.ac.kr; 4 Department of Animal Science, College of Life Science, Sangji University, Wonju 26339, Republic of Korea; meeahn20@sangji.ac.kr; 5 Biological Resources Research Group, Bioenvironmental Science and Toxicology Division, Gyeongnam Branch Institute, Korea Institute of Toxicology (KIT), Jinju 52834, Republic of Korea; jdher@kitox.re.kr; 6 T-Stem Co., Ltd., Changwon 51573, Republic of Korea

**Keywords:** flavonoid, apigetrin, cell death, apoptosis, liver cancer

## Abstract

Epidemiologic research recommends using flavonoids in the diet due to their overall health benefits. Apigetrin (Apigenin 7-O-glucoside) is a glycoside phytonutrient found in fruits and vegetables and known for different biological activities such as antioxidant and anti-inflammatory properties. Hepatocellular cancer (HCC) is a major health concern because of its adverse prognosis and side effects of chemotherapeutic agents. In the present study, we determine the impact of apigetrin on HepG2 cells and its cell death mechanism. Apigetrin reduced HepG2 cell proliferation with morphological changes and floating cells in treated cells. Colony formation and wound healing assays showed a reduced cell number in treatment groups. Further, we checked for the cell cycle through flow cytometry to understand the cell death mechanism. Apigetrin induced G2/M phase arrest in HepG2 cells by regulating Cyclin B1 and CDK1 protein levels in HepG2 cells. Annexin V and propidium iodide (PI) staining was performed to confirm the apoptotic cell population in treated groups. At the higher concentration, apigetrin showed a late apoptotic population in HepG2 cells. Chromatin condensation was also found in the treatment groups. Western blot analysis showed an increased expression of extrinsic apoptotic proteins such as FasL, Cleaved caspase 8, Cleaved caspase 3, and cleavage of PARP. In comparison, intrinsic apoptotic pathway markers showed no changes in Bax, Bcl-xL, and Cleaved caspase 9. Altogether, these findings strongly indicate that apigetrin causes cell death in HepG2 cells through the extrinsic apoptotic pathway, and that the intrinsic/mitochondrial pathway is not involved.

## 1. Introduction

Despite being the sixth most generally predominant malignant growth, liver disease is the fourth most prominent reason for cancer-related death universally [1]. Hepatitis B and C infections, fatty liver illness, liver cirrhosis, smoking, obesity, diabetes, iron overload, and other dietary practices are all risk factors [2]. Liver cancer is usually an aggressive malignancy associated with poor prognosis, and the five-year survival rate is estimated to be less than 9%. There is a need to develop naturally effective herbal compounds for the prevention and treatment of liver cancer [3]. The outcome of liver cancer is dismal. About 5% to 15% of individuals opt for surgical removal, which is only appropriate for early-stage patients with diminished hepatic regeneration ability, frequently without cirrhosis; right hepatectomy is associated with a higher risk of postoperative complications than left hepatectomy [4]. Nowadays, systemic therapy focuses on several immune-based combination medicines. Despite encouraging outcomes from a few early-phase studies, there is no documented adjuvant or neoadjuvant treatment [5].

Flavonoids are a class of bioactive components originating from plants that have been shown to have biological functions in cells [6]. Flavonoids have been recognized as crucial therapeutic adjuvants against a variety of ailments, including diabetes, arteriosclerosis, neurological disorders, and cancer. Their functions range from inflammatory modulation to cell proliferation inhibition [7]. Flavonoids have been found to have a wide range of anticancer properties, including the ability to control reactive oxygen species (ROS)-scavenging enzyme activities, engage in cell cycle arrest, induce apoptosis and autophagy, and reduce cancer cell proliferation and invasiveness [8]. Epidemiological studies have shown that consuming particular flavonoids in the diet can protect against certain cancer types [9]. Apigetrin, also known as apigenin 7-glucoside or cosmosin, is a polyphenol isolated from *Scutellaria baicalensis Georgi*, *Matricaria chamomilla*, *Stachys tibetica Vatke*, and *Teucrium gnaphalodes* [10]. Apigetrin has been demonstrated to have various pharmacological properties, including anti-melanogenesis, anticancer, hepatoprotective, and anti-inflammatory effects on the skin [11,12,13,14].

The cell cycle is a complex process involving multiple regulatory proteins that lead the cell through activities that end in mitosis and the production of two daughter cells [15]. Actively dividing cancer cells can be hindered by DNA damage, which is defined as any obstruction to DNA replication that includes broken double strands, damaged bases, or damaged nucleotides. When DNA is damaged, the cellular machinery starts DNA damage repair (DDR) in a coordinated way, resulting in either a cell cycle checkpoint, a state to enable DNA repair, or cell death when DDR intervenes or fails [16]. Kinase complexes comprise a catalytic component, the CDK (Cyclin-dependent kinase), and Cyclin, and are responsible for cell cycle regulation. CDK inhibition is likely to block cell cycle events and result in cell cycle arrest [17]. Hence, cell cycle arrest is a survival mechanism that enables tumor cells to repair damaged DNA.

Cell death is a fundamental physiological process that happens in every living organism. Its functions include embryonic development, aging, organ maintenance, im-mune response coordination, and autoimmunity [18]. Apoptosis is the process through which a cell ceases to grow and divide, instead initiating a process that eventually culmi-nates in controlled death with no leaking of its own contents into the surrounding envi-ronment [19]. Apoptosis activation results in DNA damage in precancerous lesions and can eliminate potentially hazardous cells, preventing tumor progression. Deregulating this cell death mechanism is linked to unregulated cell growth, cancer progression, and drug resistance [20].

Apoptosis pathways are divided into two major pathways: the mitochondrial (intrinsic) pathway of apoptosis and the death receptor (extrinsic) pathway. The mitochondrial apoptotic pathway results in increased mitochondrial permeability and the release of cyto-chrome c, which further binds to Apaf-1. The formation of apoptosome recruits procaspase 9, which enables downstream caspases 3, leading to apoptotic cell death [21]. The death receptor pathway occurs when a death ligand binds to a death receptor, developing DISC (disc-inducing signaling complex) and caspase 8 activation [22].

In the current study, we explored the anticancer activity of apigetrin in human hepatocellular carcinoma HepG2 cells. Apigetrin inhibited cell proliferation, G2/M cell cycle arrest, and activation of the death receptor apoptotic pathway in HepG2 cells. According to our findings, apigetrin is a possible anticancer agent in treating liver cancer cells and a nutritional source in a healthy human diet.

## 2. Materials and Methods

### 2.1. Reagent and Chemicals

The HepG2 human liver cell line was collected from the Korea cell line bank (KCLB No-88065 Seoul, Korea). Normal human liver epithelial cells (THEL2) were obtained from the American Type Cell Collection (Manassas, VA, USA). Gibco (BRL Life Technologies, Grand Island, NY, USA) supplied the fetal bovine serum (FBS), antibiotics penicillin/streptomycin (P/S), phosphate-buffered saline (PBS), and ‘Dulbecco’s modified ‘Eagle’s medium (DMEM). Sigma-Aldrich (St. Louis, MO, USA) provided propidium iodide (PI) and 3-(4,5-Dimethylthiazol-2-yl)-2,5-diphenyl tetrazolium bromide (MTT). DAPI (40, 6-Diamidino-2-phenylindole) was purchased from Vector Laboratories Inc. (Burlingame, CA, USA). Apigetrin (Purity (HPLC) >= is correct 98%) was obtained from InterPharm (KOYANG-SI, Korea). Bio-rad (Hercules, CA, USA) supplied the chemicals and electrophoresis materials. Primary antibodies against Bcl-xL (Cat. no. 2762S), Bax (cat. no. 2774S), FasL (Cat. no. 68405S), cyclin B1 (Cat. no. 12231S), caspase 3 (Cat. no. 96623), Bad (Cat. no. 9292S), cleaved caspase-8 (Cat. no. 9496S), cleaved caspase-9 (cat. no. 7237S), cleaved caspase-3 (Cat. no. 9664S), poly ADP-ribose polymerase (PARP) (Cat. no. 9542S), cleaved-PARP (Cat. no. 5625S), and β-actin (Cat. no. 3700S) were purchased from Cell Signaling Technology (Danvers, MA, USA). Secondary antibodies conjugated with horseradish peroxidase (HRP) were acquired from Bethyl Laboratories, Inc. (Montgomery, AL, USA).

### 2.2. Culture of Human Liver Cancer Cells

HepG2 cells were grown at 37 °C in a humid environment of 5% CO_2_ in a DMEM medium containing 10% fetal bovine serum (FBS) and 1% penicillin/streptomycin (P/S). Before use, apigetrin was produced using dimethyl sulfoxide (DMSO), kept at −20 °C, and diluted to the appropriate concentration with a DMEM medium. Cells were DMSO-treated or untreated with the stated dose of apigetrin for 48 h in a complete medium, and the final DMSO content was less than 0.1% in all experiments.

### 2.3. Cell Viability Assay through MTT

HepG2 cells (4 × 10^4^ cells/well) were grown for 48 h at 37 °C in 5% CO_2_. Different concentrations of apigetrin (0, 25, 50, 100, and 200 μM) were treated in the corresponding wells after 48 h; the 50 μL of 0.5% (*w*/*v*) MTT dissolved in 1× phosphate-buffered saline (PBS) was added to each well and incubated for 2 h at 37 °C in the dark. Finally, in each well 200 μL of DMSO dissolved purple formazan crystals were added. After 15 min of shaking without direct light exposure, absorbance at 540 nm was measured with a PowerWave HT microplate photometer (BioTek, Winooski, VT, USA).

### 2.4. Colony Formation Assay

HepG2 cells were seeded and incubated in 6-well plates at a (1 × 10^4^ cells/well). For 10 days, the cells were treated with the stated doses of apigetrin (0, 50, and 100 μM). The cells were fixed in 4% paraformaldehyde solution for 30 min at room temperature (RT), then stained with a Giemsa stain solution. Under a light microscope, colony development and inhibition were seen. Image J software (National Institutes of Health, Bethesda, MD, USA, 1.52a) was used to count the colonies.

### 2.5. Wound Healing Assay

HepG2 cells (4 × 10^5^ cells/well) were grown on a 6-well plate until they reached 60–70% confluence, and a straight line was drawn in the middle of each well. The wounds were cleaned with PBS and incubated for 48 h with the respective apigetrin concentrations (0, 50, and 100 μM). Image J software (National Institutes of Health, Bethesda, MD, USA, 1.52a) was used to evaluate the degree of wound healing.

### 2.6. Flow Cytometry Analysis of Cell Cycle

PI staining and flow cytometry were used to investigate the cell cycle distribution. HepG2 cells (5 × 10^5^ cells/well) were seeded in 60 mm plates and subjected to various concentrations of apigetrin (0, 50, and 100 μM) for 48 h. The cells were collected, fixed with ice-cold 70% ethanol, and stored at −20 °C and taken for analysis. After fixation, the cells were rinsed twice with cold PBS and centrifuged. The pellet was resuspended in PBS containing 50 μg/mL PI and 0.1 mg/mL RNaseA and stained for 20 min in the dark. The content was analyzed with flow cytometry on a Cytomics FC 500 (Beckman Coulter, Brea, CA, USA). In each sample, approximately 10,000 cells were sorted. The data obtained were analyzed using CXP Software (Beckman Coulter, Inc., Fullerton, CA, USA).

### 2.7. Determination of Apoptosis by Annexin V

Cell death evaluation of apigetrin-treated HepG2 cells was carried out using an allophycocyanin (APC)/Annexin V apoptosis detection kit (BD Biosciences, San Diego, CA, USA) according to the manufacturer’s protocol. A total of (5 × 10^5^ cells/well) of the cells were then exposed to the stated doses of apigetrin (0, 50, and 100 μM) for 48 h. Following centrifugation, the cells were washed with 1X PBS. The cells were resuspended in a binding buffer for 20 min in the dark before staining with Annexin V and PI at room temperature. Flow cytometry analysis was carried out after data were obtained with a FACS Calibur flow cytometer (BD Biosciences, San Jose, CA, USA).

### 2.8. Protein Extraction and Western Blot Analysis

HepG2 cells were seeded into 60 mm plates (5 × 10^5^ cells/well) and treated with apigetrin (0, 50, and 100 µM) for 48 h. RIPA lysis buffer with phosphatase and protease inhibitor cocktail was used to extract the protein. A PierceTM BCA assay was used to quantify protein concentrations (Thermo Fisher Scientific, Rockford, IL, USA). An equal quantity of protein from each sample was electrophoresed on (8–15)% SDS-polyacrylamide gels and transferred to a polyvinylidene difluoride (PVDF) membrane (ATTO Co., Ltd., Tokyo, Japan), and then the membrane was incubated with the primary antibodies followed by a conjugated secondary antibody to peroxidase. The obtained proteins were identified using an electrochemiluminescence (ECL) detection system (Bio-Rad Laboratory, Hercules, CA, USA). The densitometry readings of the protein bands were normalized by comparison with the expression of β actin as control, using the ImageJ software program (U.S. National Institutes of Health, Bethesda, MD, USA, 1.52a).

### 2.9. Statistical Analysis

The mean ± standard error of mean (SEM) of three separate experiments was used to express all of the data. Significant differences between groups were calculated using oneway factorial analysis of variance (ANOVA), followed by ‘Dunnett’s test. The *p* < 0.05, ** *p* < 0.01, *** *p* < 0.001 value was considered statistically significant.

## 3. Results

### 3.1. Apigetrin Suppresses Cell Growth and Causes Cell Death in HepG2 Cells

**Figure 1:**
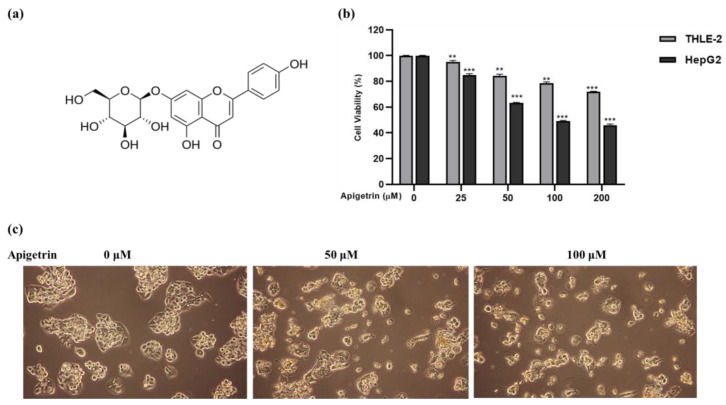
Effects of apigetrin on HepG2 cells. (**a**) Structure of apigetrin. (**b**) The cell viability effect of apigetrin on HepG2 and THEL2 cells at 48 h was measured by MTT assay with indicated concentrations (0, 25, 50, 100, and 200 µM) of apigetrin. The data are presented as the mean ± standard error of mean (SEM). ** *p* < 0.01, *** *p* < 0.001. (**c**) Morphological observations were made under a light microscope.

To examine the effects of apigetrin on cell growth, the MTT assay was carried out to determine cell viability. As shown in Figure 1a, apigetrin strongly inhibited HepG2 cells in a time- and dosage-dependent mode, whereas THEL2 cells showed no effect on cell growth after apigetrin treatment. As shown in Figure 1b, the control group’s cell shape was a fusiform or uneven polygon. The cell shape altered as apigetrin concentrations increased, and the number of detached cell counts in the top layer increased. A significant number of cells died as an effect of flocculent apoptosis.

### 3.2. Effect of Apigetrin on Cell Proliferation and Cell Migration in HepG2 Cells

**Figure 2:**
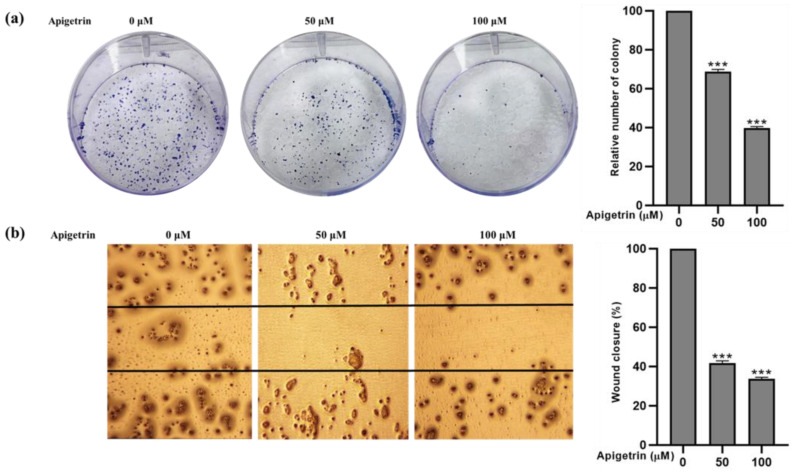
Cell proliferation and cell migration assay results. (**a**) Colony formation assay was performed on HepG2 cells at indicated concentrations of apigetrin (0, 50, and 100 µM) for 10 days and colonies were stained by Giemsa solution. (**b**) A wound healing assay was performed at stated concentrations (0, 50, and 100 µM) of apigetrin for 48 h. The data are presented as the mean ± standard error of mean (SEM). *** *p* < 0.001.

The inhibitory effects of apigetrin were validated by colony formation assay with the stated dosage (0, 50, and 100 M) for 48 h. Colony formation in HepG2 cells was inhibited significantly in apigetrin-treated groups after 10 days, confirming the cell proliferation inhibition efficiency shown in Figure 1a. Cell movement and migration were evaluated through a wound-healing assay. Increased cell migratory ability indicates cancer; the antimetastatic effect of apigetrin was analyzed by transwell assay. Figure 2b shows that apigetrin inhibited cell movement of marginal cells dose-dependently. This result outcome proves the suppression effects of apigetrin on HepG2 cells.

### 3.3. Apigetrin-Induced G2/M Phase Cell Cycle Arrest in HepG2 Cells

**Figure 3:**
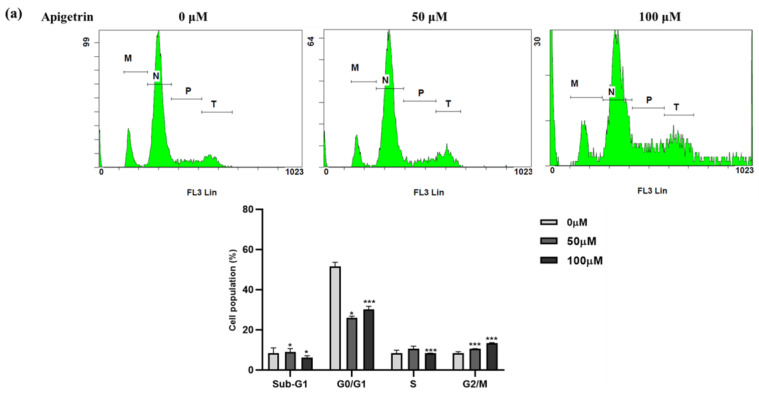

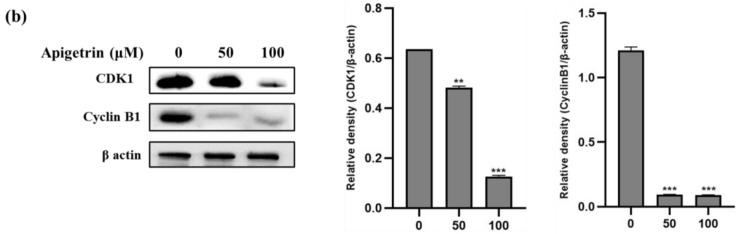
Apigetrin-induced cell cycle arrest in G2/M phase in HepG2 cells. (**a**) HepG2 cells were treated with apigetrin (0, 50, and 100 µM) for 48 h, and cell cycle states were perceived using flow cytometry. (**b**) G2/M cell cycle related proteins were identified by immunoblot. Densitometry was utilized to calculate values from three separate experiments. The data are presented as the mean ± standard error of mean (SEM). * *p* < 0.05, ** *p* < 0.01, *** *p* < 0.001.

Apigetrin could reduce cell proliferation, which fascinated our interest enough to investigate cell cycle arrest to investigate inhibitory mechanisms employing flow cytometry. According to Figure 3a, the fraction of untreated HepG2 cells in the G2/M phase of the cell cycle remained constant. In contrast, the treatment group showed a substantial rise in the G2/M phase, demonstrating cell cycle arrest. The actions of Cyclin B1 and CDK1 principally regulate the G2/M phase. We explored these proteins’ expression to understand better the molecular mechanism of apigetrin’s effect on HepG2 cell cycle arrest. The results show reduced expression of G2/M phase-related proteins (Cyclin B1 and CDK1) in a concentration-dependent mode (Figure 3b).

### 3.4. Apigetrin Caused Apoptosis and Chromatin Condensation in HepG2 Cells

**Figure 4:**
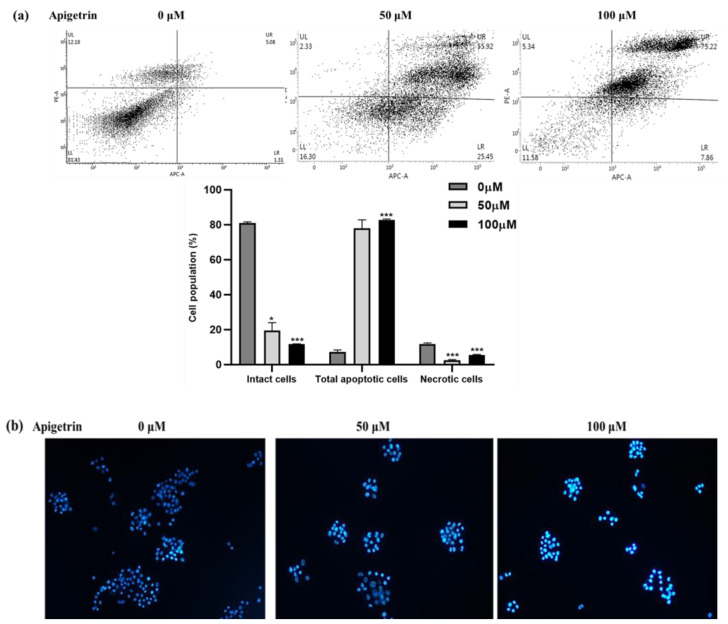
Apigetrin caused apoptosis in HepG2 cells. (**a**) To quantify apigetrin-induced apoptosis in HepG2 cells, double staining was performed at indicated concentrations (0, 50, and 100 µM) for 48 h. The data are presented as the mean ± standard error of mean (SEM) * *p* < 0.05, *** *p* < 0.001. (**b**) DAPI staining was performed to observe fragmented chromatin under a fluorescence microscope.

As shown in Figure 4a, apigetrin could increase the fraction of apoptotic cells in a concentration-dependent way. The percentage of late apoptotic cells was 55.92% at 50 μM and 75.22% at 100 μM concentrations; even the early apoptotic population was upregulated in Figure 4a. The right upper quadrant represents necrosis-related cell death; apigetrin treatment did not induce necrotic cell death and we used the DAPI assay to look at the impact of apigetrinon morphological changes and nuclear damage. Apigetrin-treated cells exhibited significant blue fluorescence, suggesting chromatin pyknosis, and blue granules, indicating cleft nuclei (Figure 4b). Our data suggest nuclear fragmentation occurs due to apoptosis in apigetrin-treated HepG2 cells.

### 3.5. Apigetrin-Induced Death Receptor-Mediated Apoptosis in HepG2 Cells

**Figure 5:**
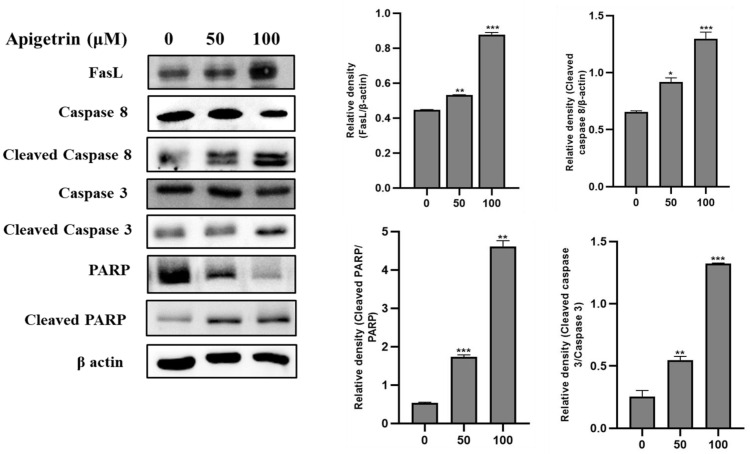
The expression of death receptor pathway proteins in HepG2 cells treated with apigetrin. Apigetrin induces Fas, FasL, cleaved caspase 8, cleaved caspase 3, and cleaved PARP protein expressions in HepG2 cells. The cells were treated with stated concentrations (0, 50, and 100 µM) of apigetrin for 48 h. Total cell lysate was resolved by sodium dodecyl sulfate (SDS)-polyacrylamide gels and transferred to polyvinylidene difluoride (PVDF) membranes. Using an electrochemiluminescence (ECL) detection process, the proteins were detected. The data are presented as the mean ± standard error of mean (SEM). * *p* < 0.05, ** *p* < 0.01, *** *p* < 0.001.

We investigated the death receptor apoptotic cell death pathway to reveal how apigetrin induces apoptosis. Immunoblot analysis revealed that apigetrin-treated HepG2 cells show apoptotic cell death through the death receptor signaling pathway. FasL (Fas Ligand), a homotrimeric protein, acts as a ligand for Fas and causes oligomerization of its receptor upon binding. Upon ligand interaction, cytoplasmic adapter proteins are recruited, which have integrating death domains that bind to the receptors. FasL expression was increased in a dose-dependent manner in HepG2 cells. Levels of cleaved caspase 8, cleaved caspase 3, and cleaved PARP showed upward trends, confirming the activation of the death receptor pathway of apoptosis (Figure 5).

### 3.6. Effects of Apigetrin on Mitochondrial-Mediated Apoptosis in HepG2 Cells

**Figure 6:**
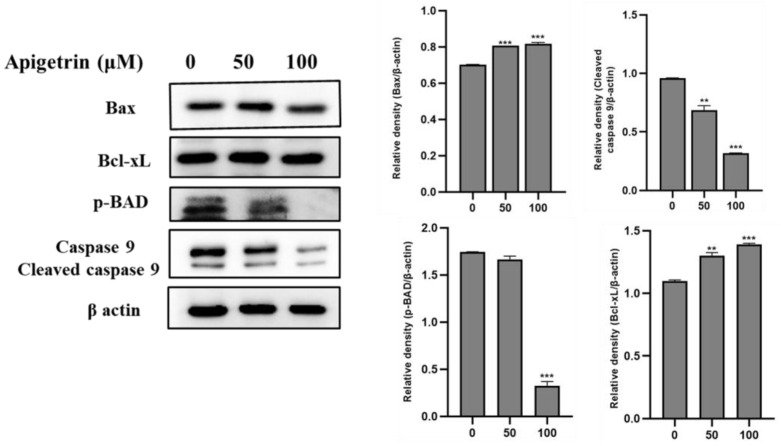
Mitochondrial signaling pathway analysis treated upon apigetrin. Apigetrin was administered to HepG2 cells at a dosage of (0, 50, and 100 µM) for 48 h. Cells were collected, and proteins were harvested by immunoblot. Bax, Bcl-xL, p-BAD, and cleaved caspase 9 were quantified by Western blot. The results obtained from three independent experiments are expressed as mean ± standard error of mean (SEM) and compared with the control group. ** *p* < 0.01, *** *p* < 0.001.

In apigetrin-treated HepG2 cells, we assessed the proapoptotic protein and anti-apoptotic protein Bax and Bcl-xL, respectively, cleaved caspase 9, and intrinsic apoptotic pathway-related proteins. As shown in Figure 6, Bax, Bcl-xL, and caspase 9 levels remained the same and cleaved caspase 9 levels decreased in apigetrin-treated HepG2 cells. P-BAD levels also decreased at the higher concentrations in HepG2 cells. P-BAD is a proapoptotic member of the Bcl-2 family that promotes cells by displacing Bax from binding to Bcl-2. These findings demonstrated that apigetrin does not promote apoptosis via the mitochondrial pathway but through the death receptor pathway.

## 4. Discussion

Natural medicine involves compounds with pharmacological or biological qualities generated naturally by living creatures such as plants, insects, animals, aquatic organisms, and microorganisms [23]. Medicinal plants remain a significant source of biologically active and therapeutically beneficial chemical entities, offering novel routes for successfully treating various human medical conditions [24]. Chamomile plants of the family Asteraceae have long been used as medicinal plants for their antioxidative and anti-inflammatory actions. Apigetrin, a major chamomile component, strongly inhibited LPS-induced NF-κB/NLRP3/caspase-1 signaling in RAW246.7 cells [25]. In another study, apigetrin caused cell death and decreased cell proliferation in gastric cancer cells by activating ROS generation, the cleavage of PARP, and the STAT3 pathway [26]. The liver cancer cell line Hep3B, when treated with apigetrin, exhibited TNFα-induced apoptosis and necroptosis through ROS generation [27]. Most anticancer mechanisms of natural compounds and chemotherapeutic medicines exert their anticancer effects by apoptosis induction and the suppression of cell proliferation on target cells [28]. Therefore, detecting apoptosis has become an important research area to determine the anticancer effects of novel medications or adjunct treatments [29].

Apigetrin effectively inhibits HepG2 cell growth in a dose-dependent manner. In this work, we have investigated the inhibitory effects of apigetrin on HepG2 cells through MTT assay. Antiproliferation and antimigration effects were evaluated by colony formation and wound healing assay. Apigetrin significantly inhibited colony formation and cell migration in HepG2 cells at 48 h.

HepG2 cells treated with apigetrin exhibited G2/M phase arrest evaluated by flow cytometry. G2/M cell cycle-related protein markers Cyclin B1 and CDK1 showed a down-regulation trend at higher concentrations in HepG2 cells. Comparable with current findings, prior studies have demonstrated that flavonoids cause G2/M arrest and apoptosis in human cancer cell lines [30,31]. Apoptosis is the most researched anticancer mechanism of natural chemicals, and chemotherapeutic therapies cause cell death by promoting apoptosis and reducing target cell development [28]. Allophycocyanin (APC)/Annexin V and PI double staining showed an increased apoptotic population in the right upper quadrant. Even the apoptotic cell death fraction in the early stage increased at higher concentrations of apigetrin-treated HepG2 cells. The molecular mechanism of apoptosis is divided into the death receptor and mitochondrial apoptotic pathways [32]. The death receptor pathway mainly regulates apoptosis by three pathways: TNFR, TRAIL, and Fas/FasL. After the death receptors are activated by their respective ligands, they start signal transduction, which leads to the recruitment of specific adaptor proteins (FADD and TRADD) [33]. Our data reveal that apigetrin treatment augmented the production of the Fas ligand (FasL), a member of the tumor necrosis factor family, and binded to Fas, causing cell apoptosis [34]. Elevated FasL further activated cleaved caspase 8, caspase 3, and polymeric adenosine diphosphate ribose (PARP) cleavage. Previously, studies exhibited similar reports about inducing death receptor apoptotic pathways on numerous cancer cells treated with flavonoids [33,35,36]. To check the involvement of the mitochondrial apoptosis pathway, expression levels of Bax, Bcl-xL, p-BAD, and cleaved caspase 9 were verified using immunoblot. The disparity between bax and Bcl-xL is shown to decide the connection of the mitochondrial apoptotic pathway [36]. Our results showed that mitochondrial protein marker levels did not show variation in HepG2 cells incubated with apigetrin treatment. Our findings could lead to further research into using innovative natural compounds in managing hepatocellular cancer.

## 5. Conclusions

**Figure 7:**
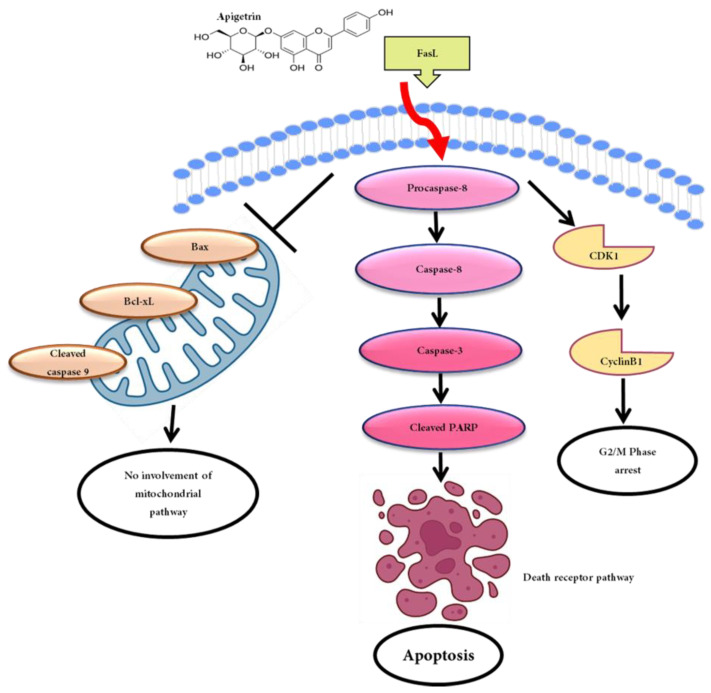
Schematic illustration of the anticancer effects of apigetrin in HepG2 cells.

In conclusion, the current research focused on the anticancer effect of apigetrin on HepG2 cells. Apigetrin could inhibit cell proliferation, induce the death receptor signaling apoptotic pathway, and cause cell cycle arrest at the G2/M phase. The anticancer effect of apigetrin on HepG2 cells occurred through the apoptotic cell death mechanism. Our findings illustrate that apigetrin promotes cell death in liver cancer cells and can be a preventive dietary component for successful adjuvant treatment. Despite this, additional clinical trials are required to verify its ability for drug development (Figure 7).

## Data Availability

The data used to support the findings of this study are available from the corresponding author upon reasonable request.
